# Antibiotic Resistance Profile and Biofilm Production of *Staphylococcus pseudintermedius* Isolated from Dogs in Thailand

**DOI:** 10.3390/ph14060592

**Published:** 2021-06-20

**Authors:** Pavarish Jantorn, Hawaree Heemmamad, Tanawan Soimala, Saowakon Indoung, Jongkon Saising, Julalak Chokpaisarn, Warapond Wanna, Varomyalin Tipmanee, Dennapa Saeloh

**Affiliations:** 1 Division of Biological Science, Faculty of Science, Prince of Songkla University, Songkhla 90110, Thailand; pavarish.j@gmail.com (P.J.); waraporn.wa@psu.ac.th (W.W.); 2 Faculty of Medical Technology, Prince of Songkla University, Songkhla 90110, Thailand; 6011510053@psu.ac.th; 3 Faculty of Veterinary Science, Prince of Songkla University, Songkhla 90110, Thailand; tanawan.soimala@hotmail.com (T.S.); saowakon.i@psu.ac.th (S.I.); 4 School of Health Science, Mae Fah Luang University, Chiang Rai 57100, Thailand; jongkon.sai@mfu.ac.th; 5 Faculty of Traditional Thai Medicine, Prince of Songkla University, Songkhla 90110, Thailand; julalak.c@psu.ac.th; 6 Department of Biomedical Sciences and Biomedical Engineering, Faculty of Medicine, Prince of Songkla University, Songkhla 90110, Thailand; tvaromya@medicine.psu.ac.th

**Keywords:** *Staphylococcus pseudintermedius*, methicillin, antibiotic resistance, biofilm

## Abstract

*Staphylococcus pseudintermedius* is a zoonotic pathogen that can cause life-threatening infections in animals and humans. The study of methicillin-resistant *S. pseudintermedius* (MRSP) and its ability to produce biofilms is important to select the most suitable treatment. The prevalence and characteristics of *S. pseudintermedius* isolated from dogs admitted at the Veterinary Teaching Hospital, Prince of Songkla University, Thailand were assessed. Results showed that 28.30% (15/53) of the isolates were MRSP. Amplification of the *mecA* gene was observed in 93.33% (14/15) MRSP. Methicillin-resistant strains revealed co-resistant patterns against other antibiotics, including chloramphenicol, clindamycin, tetracycline, clarithromycin, ciprofloxacin, and trimethoprim. In this study, all bacterial isolates produced biofilms, while 90.55% of *S. pseudintermedius* isolates were strong or moderate biofilm producers. Most (45–60%) of the resistant strains were strong biofilm producers, while the correlation between biofilm production and antibiotic resistance was not statistically significant. This is the first study in southern Thailand to investigate the drug-resistant profile of *S. pseudintermedius* and its ability to form biofilm. The results will contribute to a better understanding of the emergence and prevalence of antimicrobial resistance in *S. pseudintermedius*.

## 1. Introduction

*Staphylococcus pseudintermedius* is an opportunistic pathogen that causes wound infections, pyoderma, otitis externa, and endometritis in canines. The *Staphylococcus* intermedius group (SIG) includes *Staphylococcus intermedius*, *S. pseudintermedius*, and *Staphylococcus delphini*. *S. pseudintermedius*, found as a novel species in 2005, colonizes the skin, nose, and anus of domestic pets. SIG isolates from canine infections should be identified as *S. pseudintermedius* when tested by conventional methods [1,2]. Recently, *S. pseudintermedius* has gained increased attention, with many reports of serious invasive and opportunistic infections in humans [3]. In addition, antimicrobial resistance in *S. pseudintermedius* has also been observed, with increasing emergence of methicillin-resistant *S. pseudintermedius* (MRSP). A case report from a tertiary hospital in Sweden showed that elders were infected by MRSP, while in Thailand, *S. pseudintermedius* and MRSP were reported in pets and people who had been in contact with animals [4,5,6]. MRSP was also cultured from the grounds and exposed areas of a hospital environment [7]. *S. pseudintermedius* and MRSP were reported to show resistance to antibiotics [8,9,10], while resistance against tetracyclines, macrolides and lincosamides, chloramphenicol, aminoglycosides trimethoprim, fluoroquinolones, rifampicin, and fusidic acid was reported in *S. pseudintermedius*, with limited treatment options [11].

Biofilm is a virulence factor of *S. pseudintermedius* that promotes the adherence of bacteria to host surfaces. Biofilm formation boosts bacterial survival and growth [12,13,14]. Biofilm-related infections have been reported and investigated because the bacteria could be more tolerant to antibiotics compared to the equivalent planktonic forms and resistant to host immune responses [15,16]. Antibiotics often fail to penetrate biofilms due to the extracellular matrix, which limits the transport of antimicrobial agents. The ability of *S. pseudintermedius* to produce biofilms has been previously reported. In Japan, all *S. pseudintermedius* isolates were able to produce biofilm [17], while in South Korea, a biofilm-forming quantitative assay showed that all *S. pseudintermedius* isolates produced either strong or moderate biofilm [18].

In Thailand, the molecular epidemiology of *S. pseudintermedius* has been studied in different regions but not in southern areas [6,19]. This research assessed the prevalence and characteristics of *S. pseudintermedius* isolated from dogs admitted at the Veterinary Teaching Hospital, Prince of Songkla University, Thailand. *S. pseudintermedius* isolates were identified using a conventional biochemical method, while a genotypic method targeted the *spsL* gene. Antibiotic resistance patterns were also phenotypically and genotypically characterized, and all isolates were assessed for quantitative biofilm production. The correlation between antibiotic resistance patterns and the ability of *S. pseudintermedius* to produce biofilms was also evaluated.

## 2. Results

In this study, 53 isolates were collected from dogs admitted at the Veterinary Teaching Hospital, Prince of Songkla University. All isolates were identified using a conventional biochemical method, including a coagulase test, urease test, acetoin production test, polymyxin B resistance, and acid production from mannitol and glucose. For genotypic characterization, the PCR method was employed to confirm species of *S. pseudintermedius* by the detection of the gene encoding surface protein L (*spsL*) as a cell wall-anchored protein. A total of 53 isolates showed a positive band of 512 bp product size using gel electrophoresis. 

Detection of methicillin resistance in *S. pseudintermedius* was conducted by the Kirby–Bauer disk diffusion method. Oxacillin was chosen as a representative of all β-lactam agents owing to its stability and no longer commercially available methicillin. All *S. pseudintermedius* isolates were tested for susceptibility to oxacillin and interpretation was performed based on CLSI guidelines [20]. Results showed that 15 (28.30%) isolates were resistant to oxacillin; these were characterized as MRSP. Amplification of the *mecA* gene was observed in 14 of 15 (93.33%) MRSP isolates, while 7 (18.42%) *mecA* gene-positive isolates were found in methicillin-susceptible *S. pseudintermedius* (MSSP) (Table 1).

All isolates were tested for susceptibility to six antibiotic categories, including chloramphenicol, clindamycin, tetracycline, clarithromycin, ciprofloxacin, and trimethoprim-sulfamethoxazole, using the Kirby–Bauer disk diffusion method. Antimicrobial susceptibility testing of *S. pseudintermedius* determined that 11 (20.75%), 15 (28.30%), 15 (28.30%), 16 (30.19%), 17 (32.08%), and 20 (37.74%) isolates were resistant to chloramphenicol, trimethoprim, clarithromycin, clindamycin, ciprofloxacin, and tetracycline, respectively. Results from the disk diffusion method showed that all MRSP isolates were resistant to β-lactams and also to other antibiotics, including 8 (53.33%), 11 (73.33%), 11 (73.33%), 12 (80.00%), 14 (93.33%), and 15 (100.00%) isolates that were resistant to chloramphenicol, trimethoprim, clindamycin, clarithromycin, ciprofloxacin, and tetracycline, respectively. The oxacillin-resistant isolates were classified into seven drug-resistance patterns, as shown in Table 2. 

**Figure 1:**
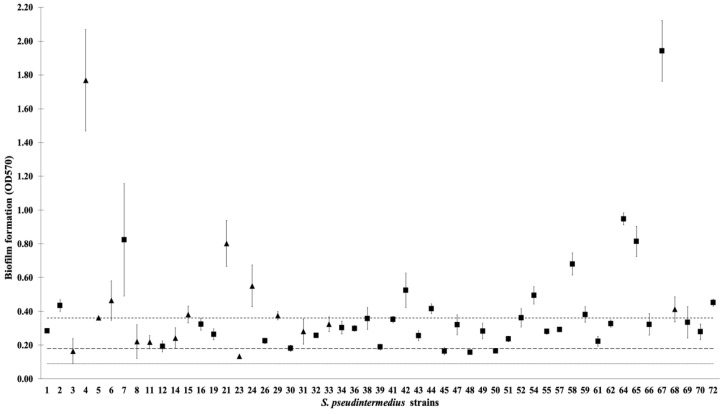
Biofilm formation values (OD570) of *S. pseudintermedius* isolates (*n* = 53) obtained by quantitative biofilm production assay. The OD cut-off used to distinguish weak and moderate biofilm producers from strong biofilm producers is 0.09 (dashed line). Categories: non-biofilm producers (OD ≤ 0.09), weak biofilm producers (0.09 < OD ≤ 0.18 (dashed line)), moderate biofilm producers (0.18 < OD ≤ 0.36 (dashed line)), and strong biofilm producers (0.36 < OD). (▲) MRSP; (■) MSSP.

In this study, all *S. pseudintermedius* isolates produced biofilms. The mean OD values obtained by a quantitative biofilm-production assay are plotted in Figure 1. Most *S. pseudintermedius* isolates showed strong and moderate production of biofilms (Table 3). Twenty-two (41.50%) isolates were identified as strong biofilm producers, 26 (49.05%) were identified as moderate biofilm producers, while only 5 (9.43%) isolates were identified as weak biofilm producers. Based on antibiotic resistance, 34.21% (13/38) MSSP isolates were classified as strong biofilm producers, 57.89% (22/38) as moderate biofilm producers, and 7.89% (3/38) as weak biofilm producers. Out of the 15 MRSP isolates, 60.00% (9/15), 26.67% (4/15), and 13.33% (2/15) were strong, moderate, and weak biofilm producers, respectively. 

Most (45–60%) of the resistant strains were strong biofilm producers, 26–45% were moderate biofilm producers, while 34–40% of susceptible strains were identified as strong biofilm producers and 50–57% were moderate biofilm producers (Table 3).

## 3. Discussion

*S. pseudintermedius* is an opportunistic pathogen that causes wound infections, pyoderma, otitis externa, and endometritis in canines. This pathogen has attracted increasing attention in recent years because invasive *S. pseudintermedius* infections have been reported in humans [3,21,22]. In this study, 53 isolates collected from dogs admitted at the Veterinary Teaching Hospital, Prince of Songkla University were identified by a conventional method. Confirmation of the strains was conducted by identifying genomic characteristics and the cell wall-anchored protein gene encoding *S. pseudintermedius* (*spsL*). Methicillin resistance is concerning because it confers resistance to all β-lactam groups. In Thailand, MRSP isolates have emerged with increasing incidence in clinical isolates, in particular, in pets and people who have been in contact with animals. A report from the Faculty of Veterinary Science Chulalongkorn University stated that 45% of *S. pseudintermedius* strains isolated from dogs between 2010 and 2012 were MRSP, while MRSP cultured from veterinarians who work there was confirmed at 76%, with 23% cultured from dog owners [6]. Phumthanakorn et al. reported 93 MRSP isolates collected from dogs (*n* = 43), humans (*n* = 18), and the environment (*n* = 32) [9]. Our study showed that MRSP was detected at 28.30%, similar to Kadlec et al., who recorded 28% MRSP isolated from dogs admitted at a small animal hospital between July 2006 and April 2013 [7]. Moreover, the Department of Dermatology at the Veterinary Hospital of Universidade Federal de Minas Gerais (Belo Horizonte, Brazil) indicated 29.55%, similar to a study at the Department of Veterinary Medicine and Animal Production, University of Naples (Naples, Italy) indicating 30.17% [23,24].

In *mecA* detection, 93.33% of the methicillin-resistant isolates contained the *mecA* gene, while 1 out of 15 isolates (6.67%) recorded an absence of the *mecA* gene. This result concurred with a previous report showing that 98.53% of oxacillin-resistant isolates were identified as carrying the *mecA* gene [25], while 9.76% of methicillin-resistant *S. aureus* (MRSA) isolates failed to detect the expression of the *mecA* gene [26]. In addition, 18.4% of methicillin-susceptible isolates contained the *mecA* gene. Chen et al. reported that 52/91 (57.1%) and 6/180 (3.3%) isolates classified as methicillin-susceptible *S. aureus* (MSSA) with oxacillin MICs were *mecA* positive [27]. In Korea, Cho et al. detected the *mecA* gene in 25 *S. pseudintermedius* isolates (19 in MRSP and 6 in MSSP isolates) [28]. Detection of the *mecA* gene was shown to be important when classifying methicillin-susceptibility testing in staphylococci, with the disk diffusion method giving high accuracy for methicillin-resistant strains. The results here suggest that antimicrobial susceptibility testing was important as well as screening for the *mecA* gene. 

The antibiotic resistance patterns were classified into seven categories by the resistant profiles. According to antibiotic classification mechanisms, including cell wall synthesis, protein synthesis inhibitors, and nucleic acid inhibitors, all resistant organisms were resistant to protein synthesis inhibitors, including chloramphenicol, clindamycin, tetracycline, and clarithromycin. Most isolates were resistant to tetracycline and protein (30s subunit) synthesis inhibitors. In *S. pseudintermedius*, tetracycline resistance was thought to be associated with producing ribosome protective protein to pump out antibiotics by an efflux system [11]. Ciprofloxacin and trimethoprim were the second and third highest resistant antibiotics. In ciprofloxacin, DNA synthesis inhibitor resistance has been reported in *S. pseudintermedius* with mutations in the *gyrA*, *gyrB*, *grlA*, and *grlB* genes, and considered resistant to all fluoroquinolones [11], while in trimethoprim, nucleic acid inhibitors were not commonly used for therapeutic interventions. The resistance was associated with an alternative insensitive target (dihydrofolate reductase) in *S. pseudintermedius* [29], probably due to the use of alternative antibiotics without knowing the need for use. Therefore, antibiotics were used excessively. Our study demonstrated antimicrobial resistance patterns of *S. pseudintermedius* isolates cultured from domestic pets. Although rarely isolated from humans, many reports detailed serious invasive and opportunistic infections in patients that may lead to cases of drug resistance *S. pseudintermedius* infection. 

Biofilms produced by *S. pseudintermedius* play an important role in the pathophysiology of infection and colonization [14,15]. In this study, all bacterial isolates produced biofilms, and more than 90% were classified as either strong or moderate biofilm producers. Meroni et al. showed that 94.5% of *S. pseudintermedius* isolates were biofilm producers, with 21 isolates strong biofilm producers, 29 moderate biofilm producers, and 19 weak biofilm producers [30]. In another study, *S. pseudintermedius* classified as strong, moderate, weak, and non-biofilm producers were 61%, 34%, 3%, and 2 %, respectively [14]. The ability to produce biofilms was different in each isolate because of several factors, such as the biofilm gene genotypic characteristic, physical interaction, and type of bacteria attachment surface. The association between biofilm production and antibiotic resistance has not been identified and correlated clearly with clonal types of drug-resistant bacteria [31,32,33]. In this study, the majority (45–60%) of the resistant strains were strong biofilm producers, with second place as moderate biofilm producers. By contrast, in susceptible strains, the majority (50–57%) were moderate biofilm producers, while strong biofilm producers were ranked below. However, variation between the OD values of resistant and susceptible strains was not statistically significant. In our study, a clear correlation was not found between antibiotic resistance and the ability to produce biofilms. 

## 4. Materials and Methods

### 4.1. Isolation of Staphylococcus pseudintermedius

Fifty-three isolates were collected from dogs admitted at the Veterinary Teaching Hospital, Prince of Songkla University (Songkhla, Thailand). All animal protocols were approved by the Institutional Animal Care and Use Committee, Prince of Songkla University (EC 2562-05-041, 7 October 2019). The bacterial strains were kept in cryogenic vials (Biologix, Shandong, China) via the glycerol (VMR International, Radnor, PA, USA) method in Luria-Bertani (LB) broth (Becton Dickinson, Le Pont de Claix Cedex, France) and stored at −20 °C. All isolates were identified using a conventional biochemical method, including a coagulase test, urease test, acetoin production test, polymyxin B resistance, and acid production from mannitol and glucose. 

### 4.2. Antimicrobial Susceptibility Testing

All isolates were grown on LB agar at 37 °C and shaken overnight. The bacteria were prepared by suspending colonies in LB broth. The log-phase bacteria were adjusted to an optical density 0.5 McFarland standard. All isolates were tested by the Kirby–Bauer disk diffusion method on Mueller-Hinton agar (Becton Dickinson, Le Pont de Claix Cedex, France) plates using oxacillin (30 μg), clarithromycin (15 μg), ciprofloxacin (5 μg), clindamycin (2 μg), trimethoprim-sulfamethoxazole (5 μg), tetracycline (30 μg), and chloramphenicol (30 μg) (OXOID, Thermo Fisher, Hampshire, UK). In this study, *S. aureus* ATCC 25923 was used as the reference strain, while all isolates were interpreted based on the Guidelines of the Clinical Laboratory and Standards Institute (CLSI, 2020) [20].

### 4.3. DNA Extraction

Pure colonies were picked from LB agar plates into 1 mL of LB broth and shaken at 37 °C overnight. The bacteria were pelleted using centrifugation at 8000× *g* for 3 min and washed three times in TE buffer. The cells were re-suspended in 500 µL of TE buffer and boiled using a heating box for 15 min. After immediate cooling on ice for 10 min, centrifugation was performed at 14,500× *g* for 10 min. The supernatant was transferred into a 1.5-mL PCR tube (Axygen, Union City, CA, USA). DNA was evaluated for purity with a NanoDrop 2000c Spectrophotometer at A260/280 (Thermo Scientific, Wilmington, DE, USA) and kept at −20 °C until required for use.

### 4.4. Gene Detection

The expression levels of *spsL* and *mecA* genes were evaluated by the polymerase chain reaction (PCR) method using a thermal cycler machine (Bio-Rad T100™ Thermal Cycler, Hercules, CA, USA). Sequences of the used primers are shown in Table 4. The reaction mixture for the PCR consisted of 2 µL of DNA extract in a total volume of 20 µL composed of 5 U/µL of *Taq* DNA polymerase (Invitrogen, San Diego, CA, USA), 10 µM of each primer, 2 mM dNTPs, 50 mM MgCl_2_, and 10X PCR buffer (Invitrogen, San Diego, CA, USA). The reaction mixture of *spsL* was thermally cycled under the following conditions: pre-incubation at 94 °C for 5 min, 25 cycles of denaturation for 30 s at 94 °C, annealing for 30 s at 60 °C, extension for 1 min at 72 °C, and final extension for 5 min at 72 °C. The PCR condition of *mecA* was set as follows: pre-incubation at 94 °C for 15 min, 30 cycles of denaturation for 30 s at 94 °C, annealing for 1 min at 59 °C, extension for 1 min at 72 °C, and final extension for 10 min at 72 °C. The purified PCR products were separated by gel electrophoresis (1.5% agarose gel (SBIO by SmartScience, Pathum Thani, Thailand) in TBE buffer (VMR International, Radnor, PA, USA)). The gel was subsequently stained with GelRed^®^ and visualized under ultraviolet light (Uvitec Ltd., Cambridge, UK).

### 4.5. Quantitative Biofilm Production Assay 

For each bacterial strain, 20 µL of 0.5 McFarland standard adjusted-bacteria were filled in 96-well flat-bottom microplates with 180 µL of tryptic soy broth (TSB) containing 1% glucose and incubated at 37 °C for 24 h. Sterile TSB containing 1% glucose was used as a negative control, while *S. aureus* ATCC 29213 was selected as a positive control. After incubation, the wells were washed three times with 300 µL of phosphate buffer saline (PBS, pH 7.2) to remove free-floating or planktonic bacteria. The wells were fixed by absolute methanol for 20 min and dried overnight. Adherence of bacteria to the culture plate was stained with 2% Hucker’s crystal violet for 15 min and excess stain was washed with distilled water. After drying, 33% (*v*/*v*) glacial acetic acid was added to the wells and the absorbance of stained adherent bacteria was measured at 570 nm using a microplate reader. The optical density (OD) value was considered as the formation of biofilm mass on the surface of the culture plate. This assay was carried out for three independent experiments in triplicate. The following criteria were used for biofilm gradation in clinical isolates. The OD cut-off (ODc) was used for biofilm gradation as three standard deviations (SD) above the mean OD of the negative control (ODc = OD average of negative control + 3×SD of OD of negative control). All strains were classified based on adherence capabilities into non-biofilm producers (OD ≤ ODc), weak biofilm producers (ODc < OD ≤ 2ODc), moderate biofilm producers (2ODc < OD ≤ 4ODc), and strong biofilm producers (4ODc < OD) [36].

### 4.6. Statistical Analysis 

Association between the antibiotic-resistant categories and quantitative biofilm production of *S. pseudintermedius* was assessed by the Chi-square test, with results expressed as means and SD of three independent experiments. Differences were considered statistically significant when *p* < 0.05. 

## 5. Conclusions

Our findings showed that *S. pseudintermedius* isolates from dogs displayed MRSP, which showed co-resistance to antibiotics. All isolates evaluated in this study could produce biofilms and this may be one of the important virulence factors in the rapidly increasing emergence of *S. pseudintermedius*. Despite the majority of resistance strains being strong biofilm producers, the correlation between antibiotic resistance and biofilm production was not statistically significant. This is the first time *S. pseudintermedius*, its drug-resistant profile, and its ability to form biofilm has been investigated in southern Thailand. The results allow us to understand more about the emergence and prevalence of antimicrobial-resistant *S. pseudintermedius*.

## Figures and Tables

**Table 1:** Comparison of methicillin susceptibility obtained by the oxacillin disk diffusion method and *mecA* gene detection.

Results Obtained by Oxacillin Disk Diffusion Method	*mecA* Gene Detection
MSSP (38/53; 71.70%)	7/38 (18.42%)
MRSP (15/53; 28.30%)	14/15 (93.33%)

MSSP: methicillin-susceptible *S. pseudintermedius*; MRSP: methicillin-resistant *S. pseudintermedius.*

**Table 2:** Antibiotic resistance profile of methicillin-resistant and methicillin-susceptible *S. pseudintermedius* isolates.

**Antibiotic Resistance Profile**	**MRSP (*n* = 15 isolates)**	
	**% Resistant**	**Number of Isolates**
C-DA-TE-CRT-CIP-SXT	20	3
C-DA-TE-CRT-CIP	6.67	1
C-DA-TE-CRT-SXT	6.67	1
C-TE-CRT-CIP	20	3
DA-TE-CRT-CIP-SXT	26.67	4
DA-TE-CIP-SXT	13.33	2
TE-CIP-SXT	6.67	1
Total	100	15
**Antibiotic Resistance Profile**	**MSSP (*n* = 38 isolates)**	
	**% Resistant**	**Number of Isolates**
C-DA-TE-CRT-CIP-SXT	2.63	1
C-DA-TE-CRT-SXT	2.63	1
C	2.63	1
DA-TE-CRT-CIP-SXT	2.63	1
DA-TE-CIP	2.63	1
DA	2.63	1
TE-SXT	2.63	1
Total	18.41	7

C: Chloramphenicol; DA: Clindamycin; TE: Tetracycline; CRT: Clarithromycin; CIP: Ciprofloxacin; SXT: Trimethoprim-sulfamethoxazole.

**Table 3:** Correlation between antibiotic resistant/susceptible strains and biofilm formation ability by the phenotypic method.

Antibiotics	% Biofilm Producer (Isolates)						*p* Value
	Resistant			Susceptible			
	Weak	Moderate	Strong	Weak	Moderate	Strong	
OX	13.33% (2)	26.67% (4)	60.00% (9)	7.89% (3)	57.89% (22)	34.21% (13)	0.12
C	9.09% (1)	45.45% (5)	45.45% (5)	9.52% (4)	50.00% (21)	40.48% (17)	0.96
DA	6.25% (1)	43.75% (7)	50.00% (8)	10.81% (4)	51.35% (19)	37.84% (14)	0.68
TE	10.00% (2)	40.00% (8)	50.00% (10)	9.09% (3)	54.55% (18)	36.36% (12)	0.58
CRT	13.33% (2)	40.00% (6)	46.67% (7)	7.89% (3)	52.63% (20)	39.47% (15)	0.66
CIP	11.76% (2)	35.29% (6)	52.94% (9)	8.33% (3)	55.56% (20)	36.11% (13)	0.39
SXT	6.67% (1)	40.00% (6)	53.33% (8)	10.53% (4)	52.63% (20)	36.84% (14)	0.54

OX: Oxacillin; C: Chloramphenicol; DA: Clindamycin; TE: Tetracycline; CRT: Clarithromycin; CIP: Ciprofloxacin; SXT: Trimethoprim-sulfamethoxazole.

**Table 4:** Primer sequences of the *spsL* and *mecA* genes used for polymerase chain reaction.

Primer Name	Sequence (5′ to 3′)	References
*spsL*-F (577 bp)	TGTGAGCGGTCAGTACGATG	[34]
*spsL*-R	CGGGAAGAAACCAGCATCGA	
*mecA*-F (162 bp)	TCCAGATTACAACTTCACCAGG	[35]
*mecA*-R	CCACTTCATATCTTGTAACG	

## Data Availability

The data presented in this study are available in article.
